# An Evaluation of Breast Augmentation: Accuracy, Utility, and Accessibility of Knowledge for Patient Education and Consultation with Artificial Intelligence

**DOI:** 10.1007/s00266-026-06063-z

**Published:** 2026-06-17

**Authors:** Emrah Işıktekin, Yusuf Furkan Kırış, Salih Kılıç, Betül Gözel

**Affiliations:** Department of Plastic, Reconstructive and Aesthetic Surgery, Faculty of Medicine, Balıkesir University, Balıkesir, Turkey

**Keywords:** ChatGPT, Breast augmentation, Breast, Artificial intelligence, Patient education

## Abstract

**Background:**

The purpose of this study is to compare the answers of the 12 most frequently asked questions regarding breast augmentation surgery on both Google Gemini and ChatGPT-4.5. Our research highlights the growing importance and differential performance of artificial intelligence models in informing patients before surgery.

**Methods:**

The 12 most asked questions about breast augmentation, based on user engagement metrics, were obtained from the Realself website. These twelve questions were investigated on both Google Gemini and ChatGPT-4.5. Information received from both platforms was analyzed and evaluated by ten plastic surgeons with European Board of Plastic Reconstructive and Aesthetic Surgery (EBOPRAS) certification. The surgeons, who reached a consensus on the application of the Global Quality Score (GQS) scale prior to evaluation, were blinded to the source of the answers. The GQS scale was used for the evaluation.

**Results:**

The average results obtained were compared with each other. While the average of Google Gemini responses was calculated at 2.842, the average of ChatGPT-4.5 responses was calculated at 3.867. For this calculation, the Wilcoxon signed-rank test was used. It was found that the ChatGPT-4.5 responses were statistically superior to Google Gemini according to the Global Quality Score (GQS) (*p* = 0.003).

**Conclusion:**

While there is emerging research on AI in plastic surgery, studies specifically comparing ChatGPT and Gemini for breast augmentation patient education using a blinded evaluation by board-certified surgeons remain limited. We suggest that AI-powered chatbots offer significant advantages for patient education but should be used cautiously. While ethical concerns persist, this study underscores the practicality of ChatGPT in informing patients about plastic surgery procedures, emphasizing the need for careful usage and collaboration to optimize benefits while minimizing risks.

**Level of Evidence V:**

This journal requires that authors assign a level of evidence to each article. For a full description of these Evidence-Based Medicine ratings, please refer to the Table of Contents or the online Instructions to Authors www.springer.com/00266.

## Introduction

One of the most performed plastic surgeries in the world is breast augmentation surgeries [1]. The increasing demand for breast augmentation surgery in society has led to the growth of the market in this field and the establishment of various Internet sites. These websites may provide useful information to people who want to have surgery before a clinical interview, as well as provide incomplete and incorrect information [2]. The answers to many of the questions about plastic surgery have been obtained for years through Google (Mountain View, CA) search [3]. Additionally, ChatGPT, which was implemented in recent history, attracted the attention of millions of people in a short time and was found to contain highly accurate information in the field of plastic surgery [4].

The aim of this study is to evaluate the answers to questions about breast augmentation and, as a result of these evaluations, to determine which AI-powered platform is capable of helping to inform patients before the examination and potentially shorten the examination time. We have identified the 12 most frequently asked questions about breast augmentation on Realself, an "online platform for patients who want to learn about plastic and aesthetic surgery procedures" with 65 million annual visits and over 8000 physician consultants [5]. These questions were posed to both Google Gemini and ChatGPT 4.5 to investigate which resource is more beneficial and adequate for patients by comparing the first answers that appeared on both platforms.

While there is emerging research on the utility of AI chatbots in plastic surgery patient education, studies specifically comparing ChatGPT and Google Gemini for breast augmentation patient education, utilizing a blinded evaluation by board-certified surgeons, remain limited. This study aims to contribute to this nascent field by providing a rigorous comparative analysis.

## Materials and Methods

The responses obtained from both platforms were assessed by ten specialists in plastic reconstructive and aesthetic surgery, all of whom held the European Board of Plastic Reconstructive and Aesthetic Surgery (EBOPRAS) qualification. Before the assessment, these surgeons achieved consensus on the interpretation and implementation of the Global Quality Scores (GQS) scale (Table 1) to ensure scoring uniformity. Throughout this assessment, the surgeons remained oblivious to one another, and the origin of the provided responses was concealed. Consequently, the evaluators were blinded to guarantee an impartial decision. Qualified specialist physicians were requested to assess the responses using a classification known as Global Quality Scores (Table 1). The mean of the total scores derived from Google Gemini and ChatGPT 4.5 was employed to assess which platform facilitates patients in obtaining more precise and beneficial information. The Wilcoxon signed-rank test was employed for this assessment.

**Table 1:** Global quality scores (GQS).

Global quality score	Description
1	Poor quality, very unlikely to be of any use to patients
2	Poor quality but some information present, of very limited use to patients
3	Some information covered but important topics missing, somewhat useful to patients
4	Good quality, most important topics covered, useful to patients
5	Excellent quality, highly useful to patients

## Result

Both Google Gemini and ChatGPT-4.5 were queried using the 12 most frequently searched questions related to breast augmentation surgery identified on the RealSelf platform. The responses generated by each artificial intelligence model were independently evaluated by 10 board-certified plastic surgeons using the Global Quality Score (GQS). The question-specific mean GQS values assigned to each platform are presented in Table 2.

**Table 2:** Average scores given by the evaluators for each question

Question	Google Gemini GQS (mean ± SD)	ChatGPT 4.5 GQS (mean ± SD)
1. What is breast augmentation?	2.60 ± 0.52	3.50 ± 0.53
2. What are the pros and cons of breast augmentation?	2.80 ± 0.42	4.00 ± 0.52
3. How should you prepare for breast augmentation surgery?	2.50 ± 0.53	4.20 ± 0.42
4. What happens during breast implant surgery?	3.60 ± 0.70	2.50 ± 0.53
5. Saline vs silicone breast implants: are they safe?	2.80 ± 0.42	3.70 ± 0.48
6. What happens during breast fat transfer?	2.70 ± 0.48	3.80 ± 0.63
7. What happens during a breast lift?	2.60 ± 0.52	4.40 ± 0.52
8. What should you expect during breast augmentation operation recovery?	3.00 ± 0.67	4.40 ± 0.50
9. What are the potential risks and side effects of breast augmentation?	2.70 ± 0.48	3.70 ± 0.68
10. When will you see breast augmentation operation result?	2.30 ± 0.48	4.00 ± 0.47
11. How long does breast augmentation last?	2.80 ± 0.42	3.80 ± 0.63
12. Can you still breastfeed after a breast augmentation?	3.70 ± 0.56	4.40 ± 0.56

For each AI platform, the scores provided by the 10 evaluators across all 12 questions were aggregated, resulting in a total of 120 individual evaluations per model. The overall mean Global Quality Score for ChatGPT-4.5 responses was calculated as 3.88 ± 0.43, whereas the mean score for Google Gemini responses was 2.78 ± 0.49, as detailed in Table 3.

**Table 3:** Summary statistics of evaluator scores

Statistic	ChatGPT 4.5	Google Gemini
Valid	12	12
Mode	4	3
Median	3.800	2.750
Mean	3.867	2.842
Std. Deviation	0.313	0.350
Coefficient of variation	0.080	0.127
Variance	0.098	0.123
Range	0.900	1.400
Minimum	3.500	2.300
Maximum	4.400	3.700

Paired statistical analyses demonstrated significantly higher GQS values for ChatGPT-4.5 compared with Google Gemini. A paired samples *t* test revealed a statistically significant difference between the two models (*t* = 4.68, *p* < 0.001). This finding was further confirmed using the Wilcoxon signed-rank test (*W* = 7.0, *p* = 0.009). The results of these statistical comparisons are summarized in Table 4.

**Table 4:** Wilcoxon signed-rank test results

Measure 1	Measure 2	W	z	p	SE Rank-Biserial Correlation
ChatGPT 4.5	Google Gemini	0	3.05	0.003	0.316

**Fig. 1:**
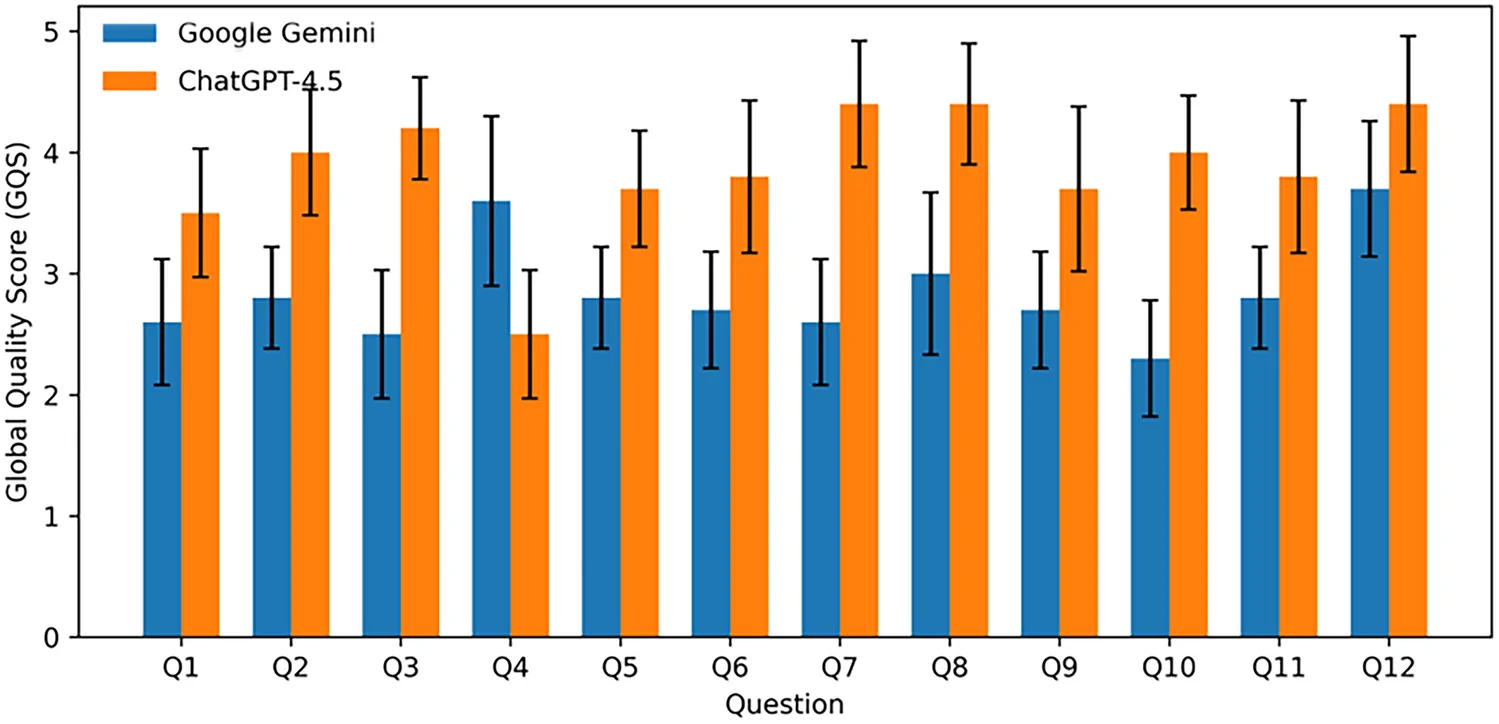
Global Quality Score (GQS) comparison between Google Gemini and ChatGPT-4.5 across twelve questions.

Effect size analysis demonstrated a very large magnitude of difference between the two platforms. Cohen’s *d* was calculated as 1.35, and the Wilcoxon effect size (*r*) was 0.72, indicating a strong effect in favor of ChatGPT-4.5. The comparative distribution of Global Quality Scores between the two AI models across the evaluated questions is illustrated in Fig. 1.

## Discussion

Aesthetic operations have always been an object of interest and curiosity for people in the past and today. Aesthetic operations have become perhaps the only option for many patients to achieve the body image they desire, and it is evident that interest in aesthetic procedures will continue to increase in the future. As technology advances, numerous innovations continue to emerge that influence both surgeons and patients. Deep learning systems have already demonstrated diagnostic capabilities comparable to fully qualified specialists in certain medical fields [6]. In parallel with these developments, artificial intelligence supported conversational platforms have gained prominence in providing information and raising awareness about surgical processes.

ChatGPT has become an increasingly utilized tool for delivering general medical information to patients considering plastic surgery. In the present study, ChatGPT, shown to be statistically superior to Google Gemini was able to provide structured, comprehensible preoperative and postoperative information related to breast augmentation. Similar observations have been reported in prior investigations, where AI-generated responses were found to be generally accurate, patient friendly, and informative, although often lacking procedural personalization [7]. Likewise, procedure-specific evaluations focusing on breast augmentation demonstrated that ChatGPT addresses common patient concerns effectively while consistently emphasizing the importance of consultation with a qualified surgeon [8]. As in our study, these findings support the role of AI as a supplementary educational resource rather than a replacement for individualized surgical counseling.

This study further demonstrates that information provided by artificial intelligence supported platforms should complement, but hopefully not replace, the information delivered by the surgeon who will perform the operation. Nevertheless, AI-supported platforms such as ChatGPT may shorten consultation times and improve patient understanding by addressing basic and frequently asked questions prior to clinical evaluation. From this perspective, such platforms may assist surgeons by enhancing baseline patient knowledge and facilitating more focused, individualized discussions during face-to-face consultations. Advances in artificial intelligence may also enable the generation of postoperative visual simulations in the future, potentially contributing to improved patient comprehension of expected outcomes and surgical goals.

Beyond its use in research and preoperative education, ChatGPT may also be utilized as a virtual health assistant. Integration of AI-supported chatbots into virtual health platforms has been proposed as a means of providing urgent perioperative assistance, reducing waiting times, and improving access to care [9]. In a prospective study involving 26 patients undergoing hip arthroscopy, a chatbot-based system was used during the postoperative period, and 80% of patients rated the chatbot as good or excellent in answering their questions [10]. These findings suggest that AI-based systems may increase healthcare efficiency by reducing the need for face-to-face consultations and supporting both physical and mental well-being. Accordingly, ChatGPT has been described as a potential “smart patient companion” [11].

However, important limitations and risks must be acknowledged. In the same hip arthroscopy study, approximately 30% of the responses generated by the AI-powered chatbot were deemed inappropriate when addressing potential health-related concerns [11]. This observation highlights the potential dangers associated with unsupervised use of AI-based medical applications [12]. Although no adverse events were reported in that particular study, these findings emphasize the necessity of careful oversight and validation. The future development of artificial intelligence in medicine will depend on continuous monitoring of AI-generated content, verification of response accuracy, and the collection of comprehensive patient data to better define system limitations [13]. With current technological capabilities, artificial intelligence supported platforms such as ChatGPT remain promising tools for delivering rational, accessible information and improving patient education prior to aesthetic procedures.

While our study provides a direct comparison between ChatGPT and Google Gemini in the context of breast augmentation patient education, it is important to acknowledge the expanding body of literature examining similar applications of AI in healthcare. A number of factors may contribute to the higher scores achieved by ChatGPT in comparison with Gemini in this study. Despite the fact that the present investigation was not designed to analyze the technical architecture of the models, it is possible that differences in training data, model maturity, and optimization for conversational responses may have contributed to the observed performance gap. ChatGPT has been extensively adopted in medical information contexts, suggesting its potential to generate more structured and patient-oriented explanations for commonly discussed procedures such as breast augmentation. Furthermore, variations in model training strategies and the extent to which each system has been exposed to publicly available medical information could influence the clarity, completeness, and organization of generated responses. Previous studies by Yun et al. (2023) [4] and Garg et al. (2024) [13] have evaluated the quality of AI chatbot responses in plastic surgery and patient education, with findings that align with our observation of ChatGPT’s superior informational performance. Additional investigations by Naufal (2025) [14] and Lombardo (2024) [15] have further explored the role of AI-generated educational materials in clinical settings, underscoring both their potential benefits and inherent limitations. Moreover, recent comparative and procedure-specific studies continue to emphasize that AI-generated medical information should be validated by clinicians and integrated cautiously into practice [16, 17].

Taken together, our findings contribute to this evolving literature by offering a blinded, surgeon-based comparison of two widely accessible AI platforms specifically in the setting of breast augmentation**.** It is important to acknowledge that the scores do not presuppose that surgeons would invariably attain a flawless evaluation. In clinical practice, there is a variability in the quality and completeness of explanations due to various factors, including the nature of the consultation, the patient's needs, and the availability of time. Therefore, the scores obtained in this study should not be interpreted as a direct comparison with an assumed ideal surgeon response but rather as an evaluation of the relative informational quality of the AI-generated answers. By preserving the central role of surgeon-led decision-making while highlighting the adjunctive value of AI-supported education, this study provides clinically relevant insight into the responsible integration of artificial intelligence into aesthetic plastic surgery practice.

## Conclusion

We demonstrated that the average score of the answers provided by ChatGPT was significantly higher than the average score of the answers provided by Google Gemini. While there is emerging research on AI in plastic surgery, studies specifically comparing ChatGPT and Gemini for breast augmentation patient education using a blinded evaluation by board-certified surgeons remain limited. In conclusion, we suggest that AI-powered chatbots offer significant advantages for patient education but should be used cautiously after assessing their risks. While ethical concerns persist, this study underscores the practicality of ChatGPT in informing patients about plastic surgery procedures, emphasizing the need for careful usage and collaboration to optimize benefits while minimizing risks.

## Declarations

### Editorial Policies

This manuscript has not been published or presented elsewhere in part or in entirety and is not under consideration by another journal. We have read and understood your journal’s policies, and we believe that neither the manuscript nor the study violates any of these. In this article, each author has equally contributed to the collection and recording of patient data. The manuscript has been read and approved by all authors.
